# The Role of Inducible Hsp70, and Other Heat Shock Proteins, in Adaptive Complex of Cold Tolerance of the Fruit Fly (*Drosophila melanogaster*)

**DOI:** 10.1371/journal.pone.0128976

**Published:** 2015-06-02

**Authors:** Tomáš Štětina, Vladimír Koštál, Jaroslava Korbelová

**Affiliations:** 1 Institute of Entomology, Biology Centre of the Czech Academy of Sciences, České Budějovice, Czech Republic; 2 Faculty of Science, University of South Bohemia, České Budějovice, Czech Republic; New Mexico State University, UNITED STATES

## Abstract

**Background:**

The ubiquitous occurrence of inducible Heat Shock Proteins (Hsps) up-regulation in response to cold-acclimation and/or to cold shock, including massive increase of *Hsp70* mRNA levels, often led to hasty interpretations of its role in the repair of cold injury expressed as protein denaturation or misfolding. So far, direct functional analyses in *Drosophila melanogaster* and other insects brought either limited or no support for such interpretations. In this paper, we analyze the cold tolerance and the expression levels of 24 different mRNA transcripts of the Hsps complex and related genes in response to cold in two strains of *D*. *melanogaster*: the wild-type and the Hsp70^-^ null mutant lacking all six copies of *Hsp70* gene.

**Principal Findings:**

We found that larvae of both strains show similar patterns of Hsps complex gene expression in response to long-term cold-acclimation and during recovery from chronic cold exposures or acute cold shocks. No transcriptional compensation for missing *Hsp70* gene was seen in Hsp70^-^ strain. The cold-induced Hsps gene expression is most probably regulated by alternative splice variants C and D of the Heat Shock Factor. The cold tolerance in Hsp70^-^ null mutants was clearly impaired only when the larvae were exposed to severe acute cold shock. No differences in mortality were found between two strains when the larvae were exposed to relatively mild doses of cold, either chronic exposures to 0°C or acute cold shocks at temperatures down to -4°C.

**Conclusions:**

The up-regulated expression of a complex of inducible Hsps genes, and *Hsp70* mRNA in particular, is tightly associated with cold-acclimation and cold exposure in *D*. *melanogaster*. Genetic elimination of *Hsp70* up-regulation response has no effect on survival of chronic exposures to 0°C or mild acute cold shocks, while it negatively affects survival after severe acute cold shocks at temperaures below -8°C.

## Introduction

Insects, as very small ectotherms with little possibility to maintain body heat, have evolved different and complex physiological mechanisms to cope with low body temperatures [1]. These mechanisms help to prevent occurrence of various types of cold injury: (a) *indirect chilling injury*: accumulates over long, chronic exposures (days to months) to mild cold (hypothermic temperatures around or above zero) and is mechanistically linked most likely with disturbed coordination of various metabolic pathways, accumulation of toxic intermediates including reactive molecules (oxidative stress), depletion of free chemical energy, and consequent disturbance of ion homeostasis [2–6]; (b) *direct chilling injury*: results from brief, acute exposures (minutes to hours) to severe cold (cryothermic temperatures close to supercooling point but without freezing), which causes dissociation of multimeric macromolecular complexes, loss of enzymatic activity, protein denaturation [7–9], phase transitions in membrane lipids, massive ion leakage, and rapid cell death [10,11]; and (c) *freezing injury*: is linked with growing ice crystals causing mechanical damage to subcellular structures, freeze-concentration of solutes that may exceed toxic limits, and severe freeze-dehydration that may cause fusions of incompatible membraneous compartments [12].

This study will focus on survival after chronic and acute exposures to cold (without freezing) in the larvae of fruit fly, *D*. *melanogaster*, an insect species with tropical origin. Tropical insects typically exhibit only limited tolerance to cold. Nevertheless, they posses clear capacity for enhancing cold tolerance in response to long-term acclimation (LTA) on a time scale of days to weeks [13–15]. In addition, practically all insects studied so far, including fruit fly, exhibited capacity to undergo very rapid adjustments of cold tolerance on a time scale of minutes to hours (so called rapid cold hardening, RCH) [16,17], which helps to prevent cold injury during diurnal changes of environmental temperature [18]. The physiological mechanisms underpinning LTA and RCH are most probably distinct [17,19]. While the RCH is primarily driven by rapid cellular processes based on signaling cascades and changes in protein phosphorylation [19], the LTA or seasonal cold acclimation is more systemic, highly complex and often triggered in advance, prior to the advent of cold season, together with transition from active life to developmental arrest called diapause [20,21]. Diapause and seasonal acclimatization represent deep phenotypic transfigurations based on global changes in gene transcription, protein expression, and metabolom composition [22–24]. The adaptive complex of LTA includes several physiological mechanisms such as regulation of activity of ice nucleators affecting the supercooling capacity [25,26], synthesis of low-molecular mass cryoprotectants [27,28], synthesis of proteins which regulate the process of ice formation [29,30], compositional remodeling of cell membranes [31], and, last but not least, up-regulation of cellular protective systems to prevent apoptosis [32], oxidative damage [33], and protein denaturation [34,35]. Due to complexity, redundancy and interplay between various processes, it is inherently difficult to study the role of individual mechanisms of LTA in separation [36].

In this paper, we assess the role of Heat Shock Proteins (Hsps), especially of the inducible form of Hsp70, in the adaptive complex of LTA in larvae of *D*. *melanogaster*. There are several good reasons for choosing the fruit fly as a model: (1) Fruit fly represents well established, genetically tractable, model organism offering good knowledge on genetic structure and physiological functions of Hsps [37–41]. (2) Fruit fly larvae possess considerable capacity to improve their cold tolerance in response to cold acclimation, both RCH and LTA [14,42,43], but the physiological basis of this phenotypic plasticity is largely unknown. (3) The species richness in the family Drosophilidae is often exploited in comparative studies on geographic clines in stress tolerance, including cold tolerance, with the aim to understand evolutionary patterns of speciation, to explain principles of ecological niche occupation, and/or to predict future responses to climate changes [44,45]. (4) The main reason, however, was that the earlier studies on the role of Hsps in the fruit fly cold tolerance brought variable results. Burton et al. [46] showed that 70 kDa Hsps were synthesized during recovery from chronic exposure to 0°C in the absence of heat shock and that a mild heat pre-treatment helped to prevent mortality caused by subsequent cold exposure. Since this pioneering study, the cold-stimulated up-regulation of Hsps complex was repeatedly confirmed in drosophilids at the levels of mRNA and proteins [47–50]. Moreover, Colinet et al. [51] reported that knocking down the expression of small *Hsp22* and *Hsp23* genes by RNAi increases chill coma recovery time. These results, together with functional (RNAi) studies performed on Hsps in other insect species [34,35], supported the view that Hsps play important role in insect cold tolerance. This view, however, was challenged in a study by Nielsen et al. [52] conducted with heat-sensitive mutant strain of *D*. *melanogaster* that harbors a mutation in the *hsf* gene that renders the gene product, heat shock transcription factor HSF, non-functional above 30°C [52,53]. Nielsen's et al. study [52] convincingly showed that HSF activation and subsequent Hsp70 expression did not occur during RCH and, although the HSF activation and Hsp70 up-regulation did occur during the LTA, no beneficial effect on fly cold tolerance was detected.

The main objective of this study was to clarify whether or not the up-regulation of inducible Hsp70 associated with LTA and recovery after cold exposure plays a role in the adaptive complex of cold tolerance of *D*. *melanogaster*. The responses to cold were compared in two fly strains: Hsp70^-^ null mutant lacking all six copies of *Hsp70* gene [54] and the wild-type strain Oregon R [55]. We focused on cold tolerance in fully grown 3rd instar larvae that were acclimated (LTA) at low temperatures (15°C followed by 6°C) in order to express their maximum cold tolerance [14]. The effects of chronic exposures to mild cold were distinguished from the effects of acute exposures to severe subzero temperatures. In addition, we used two different cold pre-treatments at sub-lethal doses of cold, again chronic or acute, in order to stimulate the expression of inducible Hsps prior to cold exposure. The expression levels of *Hsp70* mRNA transcripts and another 19 genes belonging to Hsps complex plus 4 other genes (*Frost*, *Menin*, *Cold shock protein*, and *Starvin*) potentially linked to cold acclimation/cold injury repair, were quantified by qRT-PCR but we found no compensation response for missing Hsp70 gene in Hsp70^-^ strain. We suggest that the up-regulation of *Hsp70* mRNA, which is ubiquitously observed in response to cold-acclimation and recovery after cold exposure, need not always be directly linked to, or necessary for, repair of cold injury. We observed that cold tolerance in Hsp70^-^ null mutants of *D*. *melanogaster* is compromised only when the larvae are exposed to severe cold shocks of or below -8°C. The exposures to milder doses of cold, either chronic exposures to 0°C or acute cold shocks at temperatures down to -4°C, caused similar rates of survival/mortality in the wild type larvae and the Hsp70^-^ null mutants.

## Materials and Methods

### Insects

The main experiments were conducted with two strains of *Drosophila* (*Sophophora*) *melanogaster* (Meigen, 1830): the wild-type (Oregon R strain) [55] is routinely maintained in our laboratory for decades, and *Hsp70*-null mutant strain (Hsp70^-^ strain) [54] was obtained from Bloomington Drosophila Stock Center as a stock no. 8841 with a genotype: *w*[1118]; Df(3R)Hsp70A, Df(3R)Hsp70B. The wild-type fruit fly has six nearly identical gene copies that encode Hsp70 protein and Hsp70 accounts for the bulk of Hsps that are expressed upon heat shock [56]. All six copies of *Hsp70* gene were deleted by homologous recombination in the *Hsp70*-null mutants and the Hsp70^-^ homozygous strain was established [54]. The larvae of White strain (mutation in locus *white*; [57]), which served as genetic background to create the Hsp70^-^ strain, were used in our study to verify the constitutive levels of target genes' expression in unstressed larvae. In consequence of lacking *Hsp70* gene, the Hsp70^-^ larvae and adult flies showed reduced thermotolerance, which was specifically expressed as low or almost no survival after a severe heat shock (39–39.5°C) that was preceded by a sub-lethal heat pre-treatment at 35–36°C. Interestingly, the survival after milder heat shocks (<37°C) was not affected by *Hsp70* deletion [58,59]. Stocks of all strains were maintained in glass tubes (12 cm high, 2.5 cm in diameter) at constant 18°C with 12-h/12-h light/dark (L/D) cycle in incubators MIR 154 (Sanyo Electric, Osaka, Japan). Each tube contained approximately 7–10 g of a diet containing agar (1%), sugar (5%), yeast (4%), cornmeal (8%), and methylparaben (0.2%).

In order to verify that the Bloomington stock no. 8841 lacks H*sp70* gene, we designed three different pairs of oligonucleotide primer pairs specific to *Hsp70Aa* sequence (S1 Fig) and performed the PCR amplification of genomic DNA extracted from ten adult flies of Oregon and Hsp70^-^ strains. Genomic DNA was obtained by using the DNeasy Tissue Kit (Qiagen, Hilden, Germany) in accordance to manufacturer's instructions. The PCR conditions were: T3000 cycler (Biometra, Goettingen, Germany), HS ExTaq DNA polymerase (Takara, Shiga, Japan), 20 cycles of: 30 s denaturation at 94°C, 30 s primer annealing at 61°C, and 30 s DNA extension at 72°C. The PCR products were separated on 2% agarose gel (see S1 Fig for results)

We also conducted an experiment in order to verify that the Hsp70^-^ larvae show typical pattern of reduced heat tolerance [58,59]. The larvae were collected from stock tubes and groups of approximately 20 larvae were placed on a small piece of larval diet (approximately 0.25 g) in a 2 ml plastic micro vials (the opening was closed with nylon net), which were inserted into the holes drilled into aluminum block of a dry bath incubator MD-01 (Major Science, Saratoga, California) that was pre-set to a desired temperature. The exact temperature experienced by larvae inside the plastic tubes was monitored by S0122 Temperature Logger (Comet System, Roznov pod Radhostem, Czech Republic). In the first part of this experiment, the 22°C-acclimated larvae were exposed to direct 1 h-heat shocks (HS) of variable intensity ranging from 32°C to 38°C without any pre-treatment. In the second part of experiment, the 22°C-acclimated larvae were exposed to a 1 h pre-treatment at sub-lethal high temperature of 36°C followed by severe HSs of 38°C or 39°C for 1 h. The pre-treatment and HS were always separated by 1-h-long recovery period at 22°C. After HS, the larvae were transferred on fresh larval diet in fly tubes, and held at constant 18°C for subsequent 14 d. Succesfull pupariation and emergence of fit adult flies were two criterions of survival. Exact numbers of larvae used for each specific experiment are shown in Results.

### Developmental acclimations and cold treatments

**Fig 1:**
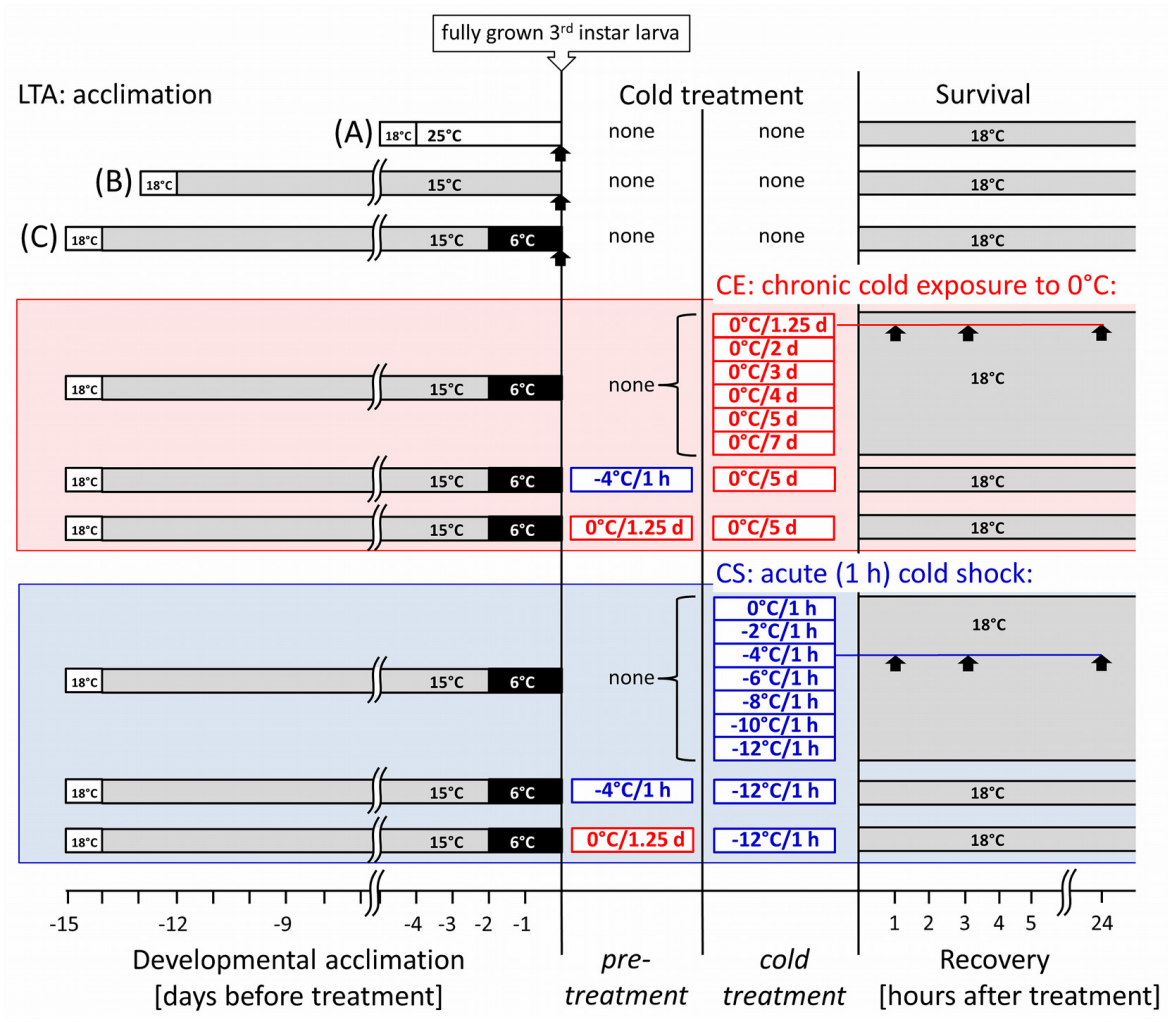
Schematic depiction of acclimation protocols and cold treatments used in this study. The experiments are represented horizontally and the main temperature and timing conditions are indicated. Vertical lines divide the experiments to four major parts: developmental acclimation, pre-treatment, cold treatment, and recovery. Larvae were acclimated under three different long-term acclimation (LTA) temperature protocols (A, B, C) and, when reaching the stage of fully grown 3rd instar, were exposed to pre-treatment [none, chronic (0°C/1.25 d), or acute (-4°C/1 h)], followed by various chronic or acute cold treatments, and recovery at constant 18°C. The pupariation and emergence of fit adults from puparia, as two criterions of survival, were checked in all experiments for 14 d of recovery. The samples for gene expression analysis were taken at time points indicated by arrows: at the end of each acclimation protocol, and on times 1, 3 and 24 h of recovery after the CE (0°C/1.25 d), or CS (-4°C/1 h).

In order to achieve developmental synchrony in fruit fly larvae, the adults (approximately 30 pairs per fly-tube) were allowed to lay the eggs during 24-long period at 18°C. The eggs were then transferred into three different temperature-acclimation protocols (see Fig 1 for schematic overview) where larval development took place (developmental acclimation *sensu* Colinet and Hoffmann, 2012). The protocols were set in incubators MIR 154 as follows:

1. constant 25°C under 12L/12D photoperiod for 4 days (25°C);
2. constant 15°C under 12L/12D photoperiod for 12 days (15°C);
3. constant 15°C under 12L/12D photoperiod for 12 days followed by 2 days at constant 6°C under constant darkness (15°C→6°C) (LTA).

All experiments were conducted with almost fully grown but still feeding 3rd instar larvae that were obtained by sampling the largest 3rd instar larvae from the diet tubes where the first wandering larvae just occurred (typically on days designated above) (this means that wandering larvae were not sampled). The acclimated larvae were then either subjected to various cold treatments or used for mRNA analysis.

In most experiments, no pre-treatment was applied. In some experiments, however, the cold exposure consisted of two steps: *cold pre-treatment* at a sub-lethal dose of cold followed by brief recovery and *cold treatment* itself. Two sorts of cold treatment were distinguished: (CE) the chronic exposure to relatively mild low temperature of 0°C for different periods of time ranging from 1.25 to 7 d; and (CS) the acute cold shock for 1 h at different low temperatures ranging from 0°C to -12°C. Similarly, the pre-treatment was either chronic (1.25 d at 0°C followed by recovery at 18°C for 2 h) or acute (1 h at -4°C followed by recovery at 18°C for 2 h). We used the term "cold pre-treatment" because our protocols differed from classic RCH protocols in duration and in the presence of recovery period during which the expression of Hsps occurred. All manipulations with larvae prior to cold treatment (or pre-treatment) were standardized in terms of time and temperature in order to minimize any potential effects on larval cold tolerance. The manipulation always took exactly 20 min and the temperature of water used to wash the larvae out of their diet was maintained same as the acclimation temperature.

The groups of approximately 20 acclimated larvae were either (CE) placed in 1 g of larval diet in a plastic tube (diameter, 1 cm; length, 5 cm), which was plugged with artificial cotton, or (CS) wrapped in between two layers of artificial cotton (75 mg) that was moistened with 300 μl of 50% glycerol in distilled water. The cotton "ball" with larvae inside was placed in the same plastic tube as above. Water in the cotton ball prevented larval dehydration during the assay, while glycerol served to prevent spontaneous crystallization of water at subzero temperatures (no freezing exotherms were observed). We verified in a preliminary experiment that glycerol had no influence on survival. The temperature inside the diet or cotton ball were continuously monitored during each experiment by using the K-type thermocouples connected to a dataloger TC-08 (Pico Technology, St. Neots, United Kingdom). For CE, the tubes with larvae were exposed to constant 0°C maintained in melting ice. For CS, the tubes with larvae were placed into the cooled bath in a programmable thermostat Ministat 240-cc (Huber, Offenburg, Germany). The temperature program comprised three steps: (i) cooling to target temperature (from 0°C to -12°C) for 10 min; (ii) maintaining the target temperature for 60 min; and (iii) heating to 0°C for 10 min.

After cold exposure, the larvae were either (CE) returned to constant 18°C inside the assay tubes that contained larval diet or (CS) unpacked from cotton balls and transferred on fresh larval diet in fly tubes. After both types of cold tolerance assays (CE and CS), the larval recovery was allowed at constant 18°C for subsequent 14 d. Succesfull pupariation and emergence of fit adult flies were two criterions of survival. Exact numbers of larvae used for each specific experiment are shown in Results.

### Analysis of mRNA transcript abundance

In total, we examined the expression of 24 different stress-related transcripts: eleven inducible *Heat shock proteins* (*Hsp70AaAb*, *Hsp 68*, *Hsp83*, *Hsp40*, *Hsp22*, *Hsp23*, *Hsp26*, *Hsp27*, *Hsp67Ba*, *Hsp67Bb*, *Hsp67Bc*), five constitutive *Heat shock cognates* (*Hsc70-1*, *Hsc70-2*, *Hsc70-3*, *Hsc70-4*, *Hsc70-5*), four splicing variants of Heat shock factor (*HsfA*, *HsfB*, *HsfC*, *HsfD*), and four other genes potentially linked to stress-response or cold acclimation/cold injury repair (*Fst*, *Mnn*, *Csp and Stv*). The S1 Table presents a complete list of target and reference genes. The relative abundances of mRNA transcripts of target genes were measured in three different experimental cohorts of larvae:

1. that were exposed to different long-term acclimations (A, B, C, see above);
2. that were acclimated at regime C (LTA), then exposed to CE of 0°C/1.25 d, and allowed to recover at constant 18°C for 1, 3 and 24 h;
3. that were acclimated at regime C (LTA), then exposed to CS of -4°C/1h, and allowed to recover at constant 18°C for 1, 3 and 24 h.

We selected the specific doses of cold exposures in CE and CS in order to ensure that larval mortality during 24-h-recovery period is small (to avoid/minimize sampling of dead animals) while the adult mortality is already clearly expressed (25–50%).

The total RNA was extracted from whole larvae (10 larvae were pooled per sample, each sample was taken in 3 biological replications) using the RiboZol RNA Extraction Reagent (Amresco, Solon, OH, USA). Pellet of total RNA was dissolved in 26 μl of DEPC-treated water and an aliquot of 1 ul was taken for total RNA concentration measurement using NanoDrop 2000 (ThermoFisher Scientific, Waltham, MA, USA). The total RNA concentrations were levelled to 1 ug/1 ul in all samples and 5 uL aliquots (5 ug of total RNA) were taken for DNase I (Ambion, Life Technologies) treatment followed by the first strand cDNA synthesis using Reverse Transcription System (Promega, Madison, WI, USA). The cDNA products (20 μL) were diluted 25x with sterile water and used as templates for qRT-PCR reactions.

Relative abundances of mRNA transcripts for target genes were measured by quantitative Real Time PCR (qRT-PCR) using the CFX96 PCR light cycler (BioRad, Philadelphia, PA, USA) and the IQ SYBR Green SuperMix (Bio-Rad). PCR reactions were primed with a pair of gene-specific oligonucleotide primers (S1 Table). Special care was taken to design the primers that are highly specific to individual genes belonging to structurally similar family (*i*.*e*. family of six *Hsp70*, *Hsp68*, *Hsp83* and five *Hscs*, family of seven small *Hsp*s, four splicing variants of *Hsf*). The PCR products of all primer pairs were sequenced (GATC Biotech, Constance, Germany) and the sequences were BLASTed (blastn) against NCBI database. In all cases, the sequence of PCR products matched almost perfectly with the respective target gene. Two genes, *Hsp67Bb* and *Hsp22*, however show overlapping sequences in some alternative mRNA transcripts (See S1 Table for details). In this case, our results do not assure perfect specificity but we decided to keep the data in the paper because the gene expression patterns of two genes differed. In the case of *Hsp70* gene, our qRT-PCR primer pair was highly specific for gene copies *Aa* and *Ab*. Therefore, we use the abbreviation *Hsp70Aa*, *Ab* for its mRNA. Emission of a fluorescent signal resulting from SYBR Green binding to double-stranded DNA PCR products was detected with increasing PCR cycle number. Quantitation cycle (*C*_Q_) for each sample was automatically calculated using the algorithm built in the CFX96 PCR light cycler software. The levels of mRNA transcripts of *Ribosomal protein L32* and *β-tubulin 56D* (S2 Fig) served as endogenous reference standards for relative quantification of the target transcript levels [60]. Each sample was run as a doublet (two technical replicates) of which the mean was taken for calculation.

### Statistics

A lethal *time* or *temperature* to kill 50% of a population sample (*Ltime*_*50*_ or *LTemp*_*50*_, respectively) was calculated for CE or CS treatments, respectively, from sigmoidal dose-response survival curves as an inflection point between Top (calculated from curve) and Bottom (constrained to 0). The differences in survival between two fly strains (Oregon and Hsp70^-^) were then assessed using Student's unpaired two-tailed t-tests (alpha = 0.05) or using analysis of contingency tables (Chi-square test). Relative ratios of the target mRNA levels (*C*_Q_) to geometric mean of the levels (*C*_Q_) of two reference gene mRNAs were calculated according to Pfaffl [61]. The Log2-transformed relative ratios were statistically analyzed using one-way ANOVA (with confidence intervals set to 95%) followed by Bonferroni post-hoc tests or, where only two means were compared, using Student's unpaired two-tailed t-tests (alpha = 0.05). The above statistical calculations were performed using Prism6 (GraphPad Software, San Diego, CA, USA). The global trends in target gene expression were analyzed using Principal component analysis (PCA) with Canoco v. 4.52 (Biometris-Plant Research Institute, Wageningen, The Netherlands).

## Results

### The larvae of Hsp70^-^ strain show impaired heat tolerance

**Fig 2:**
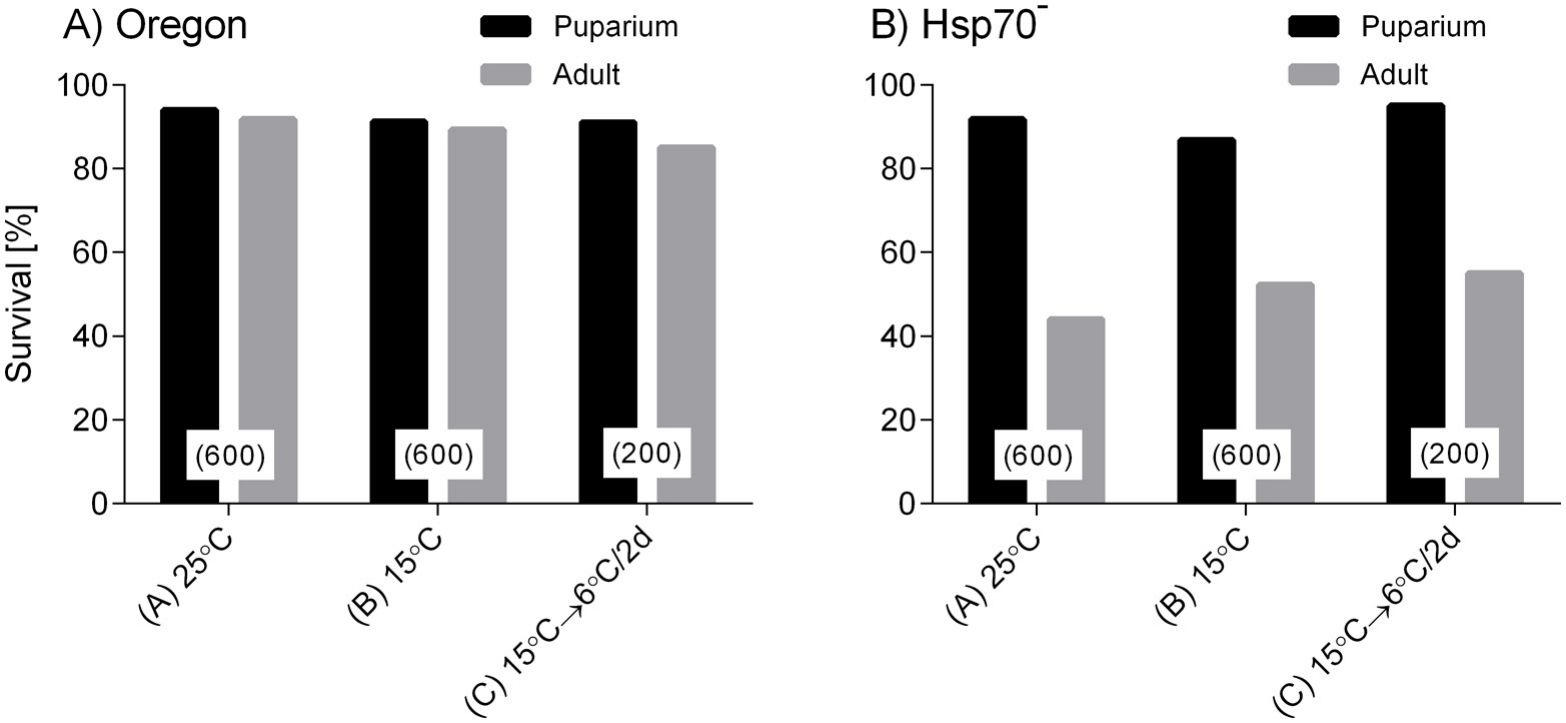
Survival in *Drosophila melanogaster* larvae of two strains, Oregon and Hsp70^-^, when exposed to different long-term acclimations (A, B, C). Black columns show proportion of individuals that were able to form puparium and grey columns show proportion of individuals that finally emerged as fit adults. Numbers (*N*) of larvae in each acclimation/ strain treatment are shown in parentheses.

Fig 2 shows survival in larvae of Oregon and Hsp70^-^ strains exposed to different long-term acclimations (A, B, C). Under all acclimation regimes, larvae of both strains showed high survival until pupariation (ranging between 86.8–95%). Two strains, however, differed dramatically in their survival until the adult fly stage. In Oregon strain, majority of pupariated larvae always produced fit adults (overall survival to adults ranged between 89.3–91.8%). In contrast, we typically observed relatively high mortality occurring between puparial and adult stages in Hsp70^-^ strain where final survival to adult stage ranged only between 44.0–55.0%. The mortality was expressed most often as inability of pharate adults to open puparial case or to leave the puparium, or as morphological abnormalities in eclosed adult flies (most often insufficiently inflated wings). Therefore, in order to avoid distortion of our results by constitutively high pharate adult mortality in Hsp70^-^ strain, we used the survival until puparial stage as the main between-strain comparative criterion of survival in heat and cold tolerance experiments.

**Fig 3:**
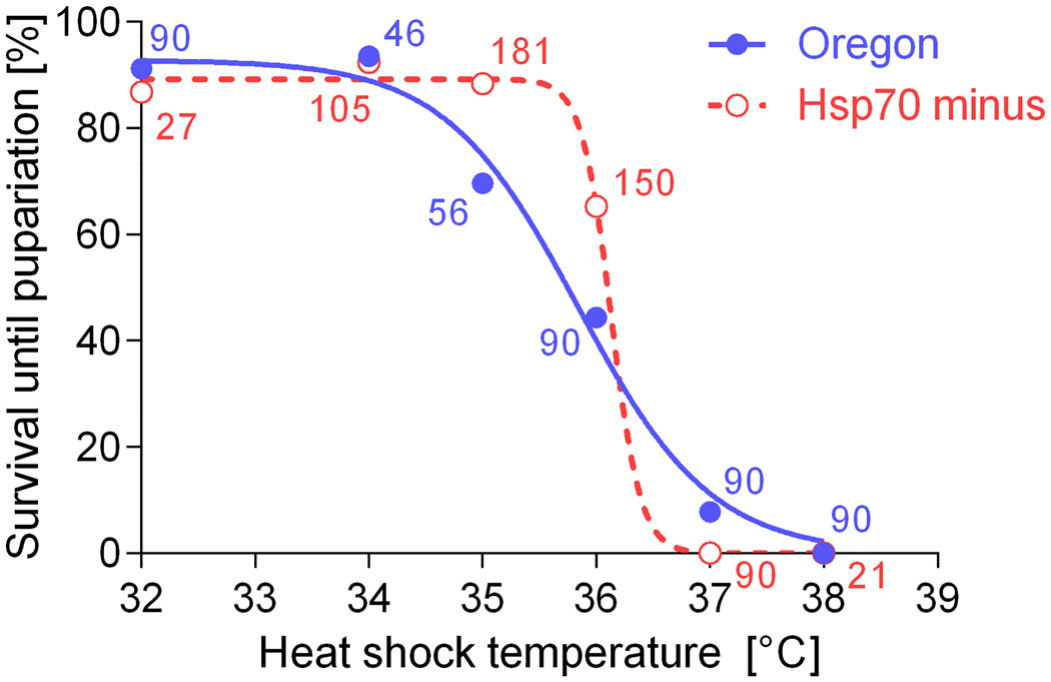
Survival in *Drosophila melanogaster* larvae of two strains, Oregon (blue full circles) and Hsp70^-^ (red empty circles) acclimated at constant 22°C and then exposed to 1 h heat shocks of variable intensity ranging from +32°C to +38°C. Numbers flanking each data point show numbers (*N*) of larvae in each experiment. Sigmoid survival curves were fitted to data (goodness of fit, R^**2**^: 0.9895, Oregon; 0.9983, Hsp70^-^).

Fig 3 shows results of heat tolerance assays. We found very little difference in survival between Oregon and Hsp70^-^ larvae that were subjected to direct 1 h-long HSs of different intensity (varying from 32°C to 38°C) without any pre-treatment. The *LTemp*_*50*_ values were practically equal in two strains: Oregon, 35.84°C; Hsp70^-^, 36.13°C (t-test: *t* = 1.796, *df* = 10, *P* = 0.1028). No larva survived when subjected to a direct HS of 38°C. When a brief 1 h-pre-treatment at 36°C was applied prior to the main HS, the survival was positively affected in Oregon strain larvae: 60.6% (*N* = 160) of pre-treated larvae survived at 38°C and 38.3% (*N* = 120) survived at 39°C (these results are not included in Fig 3). In contrast, very small or no positive effect of pre-treatment was observed in the Hsp70^-^ strain larvae: 7.5% (*N* = 160) of pre-treated larvae survived at 38°C and 0.3% (*N* = 120) survived at 39°C. These results confirmed that the induced heat tolerance is significantly impaired in Hsp70^-^ strain in comparison to Oregon strain larvae.

### The larvae of Oregon and Hsp70^-^ strains do not differ in survival after chronic exposure to 0°C

**Fig 4:**
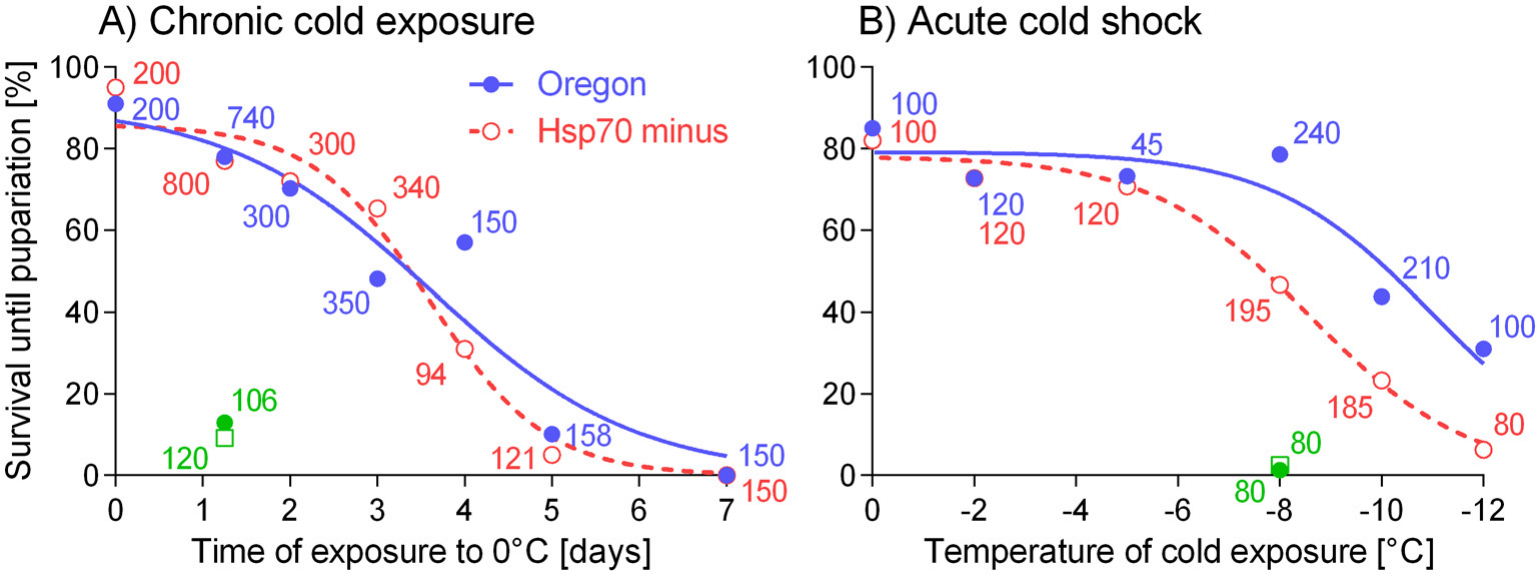
Survival in *Drosophila melanogaster* larvae of two strains, Oregon (blue full circles) and Hsp70^-^ (red empty circles) acclimated under protocol C (15°C followed by 6°C/2 d) and then exposed to (A) chronic cold exposures (0°C), or (B) acute (1 h) cold shocks of variable intensity ranging from (A) 0 d to 7 d, or (B) 0°C to -12°C. Numbers flanking each data point show numbers (*N*) of larvae in each experiment. Sigmoid survival curves were fitted to data [goodness of fit, R^**2**^: (A) 0.9124, Oregon; 0.9743, Hsp70^-^; (B) 0.8881, Oregon; 0.9916, Hsp70^-^]. Green symbols show analogous data collected for larvae that were acclimated under protocol B (15°C constant).

Fig 4A shows that the *Ltime*_*50*_ values were practically equal in two strains: Oregon, 3.61 d; Hsp70^-^, 3.63 d (t-test: *t* = 0.07717, *df* = 10, *P* = 0.9400). Only a small proportion of larvae survived until pupariation after the CE of 5 days (Oregon, 10.1%; Hsp70^-^, 5.0%) and none survived after the CE of 7 days. No positive effect on survival was observed when we applied the chronic or acute pre-treatments prior to CE of 5 days. In contrast, the pre-treatments had weak negative effects on survival, which means that the control (not-pretreated) larvae had slightly lower risks of death caused by CE of 5 days than the pre-treated larvae (Table 1).

**Table 1:** The effect of cold pre-treatments on survival of *Drosophila melanogaster* larvae after the chronic cold exposure (CE) to 0°C for 5 days.

Strain	pre-treatment ^1^	(*N*)	pupariatedlarvae	deadlarvae	*P* ^2^	Relative risk of death ^3^ [%]
Oregon	no	(158)	16	142		
	acute	(60)	2	58	0.1657 ns	92.97
	chronic	(110)	4	106	0.0584 ns	93.26
Hsp70^-^	no	(121)	6	115		
	acute	(60)	0	60	0.1802 ns	95.04
	chronic	(60)	0	60	0.1802 ns	95.04

^1^ Acute pre-treatment, 1 h at -4°C; chronic pre-treatment, 1.25 d at 0°C. Both pre-treatments were followed by recovery at 18°C for 2 h.

^2^ Statistical significance of the difference between control (no pre-treatment) and pre-treated larvae was tested using Fishers' exact test. Significant differences are shown in bold letters: *, *P* < 0.05; ns, not significant.

^3^ The relative risk of death expresses the chance of death in control larvae (not pre-treated) in comparison to pre-treated larvae.

### The larvae of Hsp70^-^ strain show impaired survival after severe acute cold shock

Fig 4B shows that the *LTemp*_*50*_ values highly significantly differed between two strains: Oregon, -11.00°C; Hsp70^-^, -8.59°C (t-test: *t* = 8.668, *df* = 10, *P* < 0.0001). Only 46.6, 23.3 and 6.3% of Hsp70^-^ larvae survived until pupariation after the severe CSs of -8°C, -10°C or 12°C respectively); while 78.6, 43.8 and 31.0% (respectively) of Oregon larvae survived the same treatments. No positive effect was observed when we applied either chronic or acute pre-treatments prior to severe CS of -12°C. The negative effect of pre-treatments on survival was highly statistically significant in the case of Oregon strain larvae, while it is was not significant in Hsp70^-^ larvae (Table 2).

**Table 2:** The effect of cold pre-treatment on survival of *Drosophila melanogaster* larvae after the acute cold shock (CS) to -12°C for 1 hour.

Strain	pre-treatment ^1^	(*N*)	pupariatedlarvae	deadlarvae	*P* ^2^	Relative risk of death ^3^ [%]
Oregon	no	(100)	31	69		
	acute	(100)	10	90	0.0004 ***	76.67
	chronic	(65)	7	58	0.0025 **	77.33
Hsp70^-^	no	(80)	5	75		
	acute	(100)	2	98	0.2442 ns	95.66
	chronic	(50)	4	46	0.7325 ns	100.02

^1^ Acute pre-treatment, 1 h at -4°C; chronic pre-treatment, 1.25 d at 0°C. Both pre-treatments were followed by recovery at 18°C for 2 h.

^2^ Statistical significance of the difference between control (no pre-treatment) and pre-treated larvae was tested using Fishers' exact test. Significant differences are shown in bold letters:

**, *P* < 0.01;

***, *P* < 0.001; ns, not significant.

^3^ The relative risk of death expresses the chance of death in control larvae (not pre-treated) in comparison to pre-treated larvae.

### The larvae of three different strains differ in constitutive levels of expression of target genes

The S3 Fig summarizes the constitutive differences in relative levels of target gene mRNAs in the unstressed (25°C-acclimated) larvae of three different strains. Considering a two-fold difference as a threshold, ten genes were constitutively up-regulated in the Hsp70^-^ strain larvae when compared to Oregon strain larvae: *Hsp67Bb* (5.6-fold), *Hsp23*, *Hsp68*, *Fst*, *Hsp67Bc*, *Hsc70-4*, *Hsp40*, *Hsp22*, *Hsp67Ba*, *and Hsc70-5* (2.0-fold). None of the target genes was statistically significantly down-regulated in Hsp70^-^ larvae, though the transcript levels of *Hsp27* were 1.6-fold lower in Hsp70^-^ larvae than in Oregon larvae (S3A Fig). The observed constitutive up-regulation of target genes in Hsp70^-^ strain was probably linked to genetic background of White strain because a group of similar ten genes were constitutively up-regulated in the White strain larvae when compared to Oregon larvae: *Hsp67Bb* (3.5-fold), *Hsp23*, *Mnn*, *Hsp68*, *Hsp67Bc*, *Fst*, *Hsp67Ba*, *Hsp22*, *Hsc70-1*, *and Hsp26* (2.1-fold). The transcript levels of *Hsp27* were 1.9-fold lower in White larvae than in Oregon larvae. In addition, we found that the levels of *Hsp70Aa*, *Ab* transcripts were also constitutively 3.5-fold up-regulated in White strain compared to Oregon strain (S3B Fig).

### Gene expression responses to long-term cold-acclimation do not differ between two strains

The responses to three acclimation regimes (A, B, C) were quantitatively and qualitatively similar in the larvae or Oregon and Hsp70^-^ strains with one obvious exception: missing *Hsp70* mRNA transcripts in Hsp70^-^ strain. In Oregon strain, the levels of *Hsp70Aa*, *Ab* mRNA were significantly up-regulated 14.7-fold in cold-acclimated larvae (C) when compared to pre-acclimation levels (A). It is obvious that this up-regulation occurred mainly in response to the final step of LTA in regime C (*i*.*e*. in response to 6°C/2 d) (S4 Fig).

**Fig 5:**
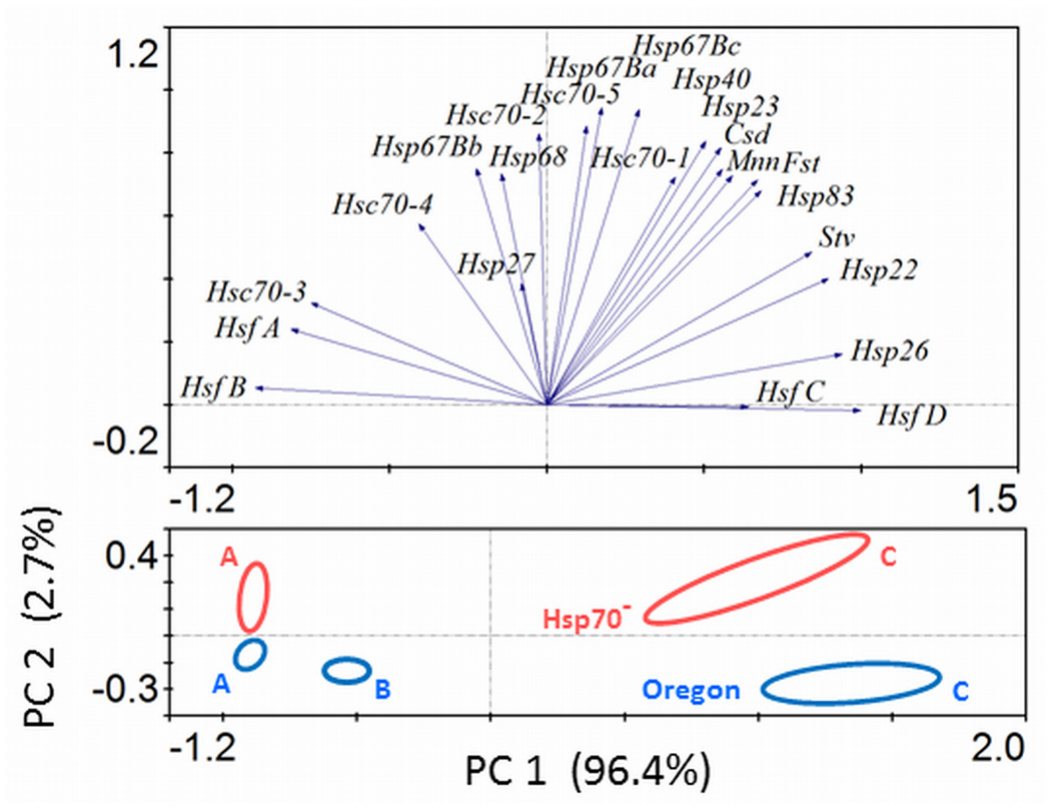
Gene expression response to different long-term acclimations (A, B, C) analyzed by Principal Component Analysis (PCA) in *Drosophila melanogaster* larvae of two strains, Oregon (blue symbols) and Hsp70^-^ (red symbols). Log2-transformed values of the fold-differences in relative mRNA levels (shown in S4 Fig) were fitted into the PCA model and a plot of principal components PC1 and PC2 is presented. The ellipsoids in lower part of Fig 5 delimit the areas of clustering of three biological replications of each treatment. The eigenvectors in the upper part of Fig 5 represent individual mRNAs.

The general patterns of gene expression to acclimation regimes were assessed using PCA (Fig 5) and detailed results are summarized in S4 Fig In order to avoid serious distortion of PCA results due to missing *Hsp70* mRNA transcripts in Hsp70^-^ strain, we excluded *Hsp70* mRNA from PCA. The difference in constitutive levels of target gene expression between two strains is apparent as a separation of the respective samples A according to the PC2 component. Three acclimation regimes are very clearly separated according to PC1 component which accounts for 96.4% of total inertia (Fig 5).

The eigenvectors representing individual mRNAs are shown in the upper part of Fig 5. The eigenvectors can be used to identify major driving forces of the overall changes in gene expression. By far the highest acclimation-related up-regulation was seen in *HsfD* mRNA: 82-fold in Oregon, 93-fold in Hsp70^-^ strain. The acclimation-related up-regulation responses of lower magnitude (up to 6-fold) were observed, in both strains, also in *HsfC*, *Hsp22*, *Hsp26*, *Fst* and *Stv*. All small *Hsp*s and also *Mnn* showed a similar two-step response, where the levels were initially down-regulated in response to acclimation at 15°C, while they were subsequently up-regulated in response to final step of 6°C/2 d (see S4 Fig). A down-regulation of mRNA levels in response to cold-acclimation was very clearly expressed in *HsfA*: 201-fold in Oregon, 139-fold in Hsp70^-^ strain, and also in *HsfB* and *Hsc70-3*.

### Gene expression during recovery: no compensation for missing *Hsp70* gene in Hsp70^-^ strain

**Fig 6:**
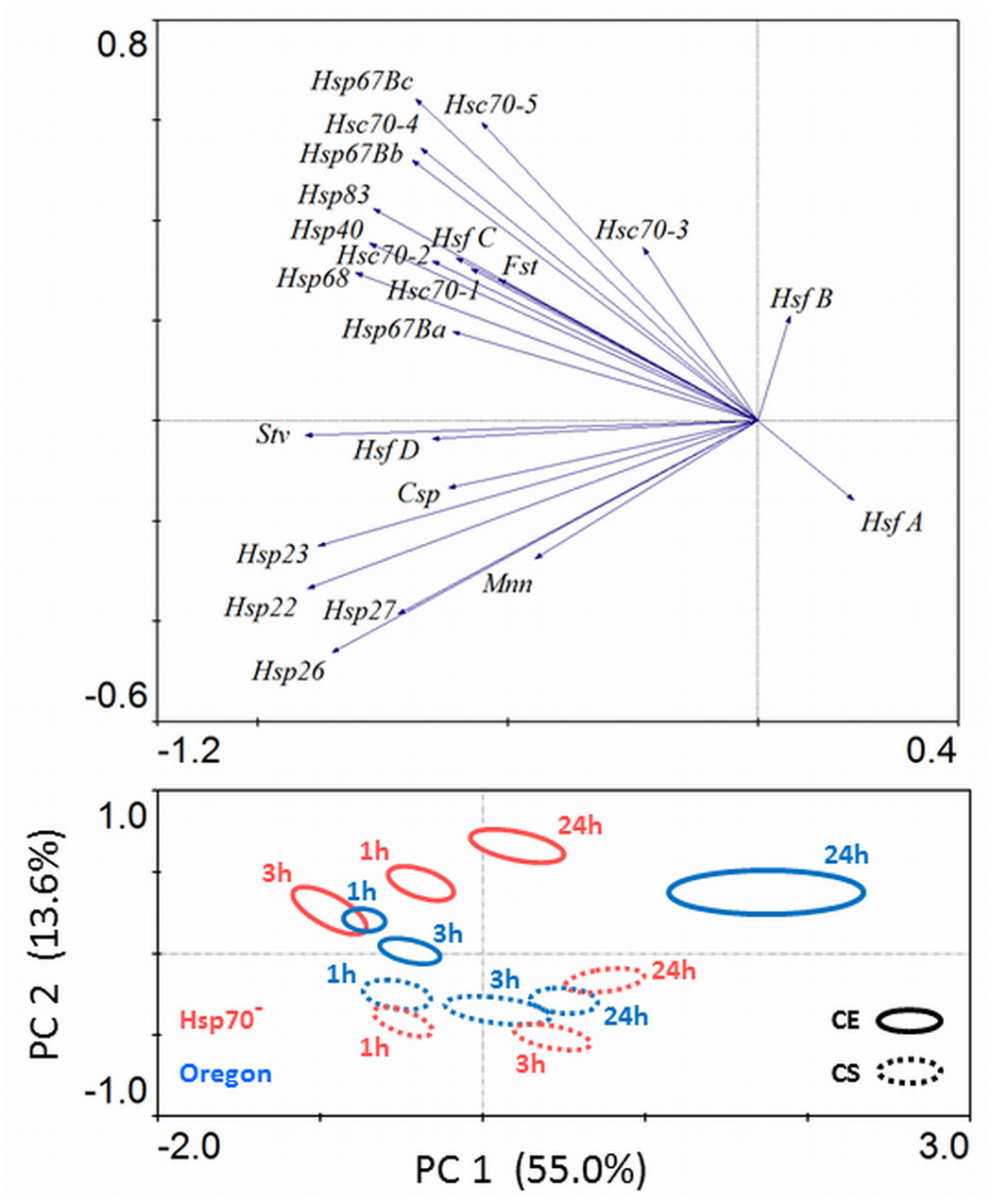
Gene expression response to recovery from chronic cold exposure (CE, solid lines) or cold shock (CS, dashed lines) analyzed by Principal Component Analysis (PCA) in *Drosophila melanogaster* larvae of two strains, Oregon (blue symbols) and Hsp70^-^ (red symbols). Log2-transformed values of the fold-differences in relative mRNA levels (shown in S4 Fig) were fitted into the PCA model and a plot of principal components PC1 and PC2 is presented. The ellipsoids in lower part of Fig 6 delimit the areas of clustering of three biological replications of each sample. Samples were taken at three different times (1h, 3h, 24h) of recovery at constant 18°C. The eigenvectors in the upper part of Fig 6 represent individual mRNAs.

The mRNA levels of inducible *Hsp70Aa*, *Ab* were again excluded from PCA analysis (Fig 6) because the *Hsp70A* gene was missing in Hsp70^-^ strain, while it was strongly up-regulated during recovery after cold treatment in Oregon strain larvae. The highest levels of *Hsp70Aa*, *Ab* mRNA in Oregon strain were observed at 1 h of recovery from chronic exposure (CE): 18.7-fold higher in comparison to cold acclimation (C); or 275-fold higher in comparison to pre-acclimation (A). At 1 h of recovery from acute cold shock (CS), the *Hsp70Aa*, *Ab* mRNA levels were 3.2-fold higher in comparison to (C); or 47-fold higher in comparison to (A) (S4 Fig).

Four principal components of PCA explained the variance in mRNA transcript abundances as follows: PC1, 55.0%; PC2, 13.6%; PC3, 9.6%; and PC4, 7.7% (85.9% in total). The plot of the first two PC components (Fig 6) shows clear separation of samples taken at different times of recovery according to PC1 component, which probably describes the rapid temporal variation that is characteristic for inducible Hsps' gene expression. The PC2 component separates the samples exposed to chronic cold (CE, positive values of PC2) from samples exposed to acute shock (CS, negative values of PC2). Two different strains of *D*. *melanogaster* are not apparently separated in the PCA plot.

Except the obvious between-strain difference in *Hsp70* gene expression, the other genes behaved similarly in both strains. In no single case (no gene) we observed considerable up-regulation of other gene that would compensate for missing *Hsp70* gene in Hsp70^-^ strain. In both strains, similar up-regulations during recovery from cold were observed in all inducible Heat shock protein genes and in *Stv* (S4 Fig). PCA suggests that small inducible *Hsps 22*, *23*, *26* and *27* are good biomarkers of the CS-response, while the large *Hsps 40*, *68*, *83* and small *Hsps 67b*, *c* are good biomarkers of the CE-response (Fig 6).

## Discussion

### Up-regulation of *Hsp70* mRNA transcripts in response to cold is not always needed for the repair of chill injury

In this paper, we show that different low-temperature treatments stimulate considerable up-regulation of inducible *Hsp70* mRNA transcripts in the wild-type larvae of *D*.*melanogaster*. This finding is not novel. In fact, stimulation of Hsp70 expression in response to cold was often observed not only in *D*. *melanogaster* [47–50] but in many other insects as well [62–65]. Although functional assays were seldom conducted, the ubiquitous occurrence of massive Hsp70 up-regulation led to speculations about its direct involvement in the repair of cold injury. RNAi was used to suppress the Hsp70 up-regulation response to cold in two insects: pupae of *Sarcophaga crassipalpis* [34] and adults of *Pyrrhocoris apterus* [35]. In both cases, the RNAi suppression of *Hsp70* resulted in decreased cold tolerance. Similarly, RNAi suppression of small *Hsp*s *22* and *23* resulted in impaired recovery from cold treatment in the adults of *D*.*melanogaster* [51]. The effect of RNAi was most significant in *S*. *crassipalpis* where the survival of diapausing pupae after cold shock of -15°C/24 h was reduced by injection of *Hsp70* dsRNA from 80% in controls to ca. 15% in young individuals or to ca. 50% in older individuals [34]. In *P*. *apterus*, the effect of Hsp70 suppression was relatively small, mainly affecting the proportion of insects that were not able to fully repair cold injury (uncoordinated crawling) but remained alive after cold treatment [35]. These results strongly supported significant contribution of the Hsps' up-regulation in the repair of cold injury. However, the cold tolerance of RNAi-treated insects was not lost completely but rather decreased partially in comparison to untreated controls, which corresponds well with general view that cold tolerance represents whole complex of physiological adjustments [17,19] of which Hsps upregulation is only one element. The relative importance of this element will strongly depend on species and population (taxonomic context), ontogenetic stage of development, diapause and acclimation status (physiological context), and exact environmental conditions (ecological context).

One of the well-established roles for inducible Hsps in *D*. *melanogaster* is to facilitate heat tolerance [37–41, 56]. Especially the larvae, which are restricted to sunlit necrotic fruits, are regularly exposed to very high temperatures that occasionally cause their mortality [56]. Heat stress typically disrupts the native conformation of proteins [66]. Denatured proteins expose their hydrophobic side-groups, which then cause aggregation, lack of functional proteins and direct toxicity. In this situation, Hsps function as molecular chaperones. They recognize and bind denatured proteins, shield the hydrophobic residues from inappropriate interactions and, later, help re-folding the denatured proteins or target them for degradation [67]. In addition, heat shock cognates play similar roles in the absence of stress. They assist during folding of nascent peptides, or participate during transport of unfolded proteins across membranes [56]. Whole range of other stress factors, including cold, can disturb normal process of nascent protein folding and protein transport, cause dissociation of polymeric proteins and/or partial denaturation of proteins [7,8]. That is why the up-regulation of inducible Hsps is so often observed in direct response to cold. Nevertheless, many cold-dissociated protein complexes are able to re-assemble spontaneously upon re-warming [7,8]. Therefore, it may happen that exposure to relatively weak dose of cold will cause some up-regulation of inducible Hsps, but this will have little or no observable effect on cold tolerance. Other injuries linked to cold exposure, such as membrane failure, might be much more important and their occurrence and scope will then decide about survival/mortality. It is also important to note that the amount of inducible Hsp70 protein produced after temperature shock is always less than the constitutively synthesized level of cognate Hsps [68]. Therefore, cognate forms can significantly contribute to cold tolerance (repair of direct chilling injuries) irrespective of presence/absence of the inducible Hsps forms. Cognates and various inducible Hsps act redundantly in the repair of cold injury and may mutually compensate for functions of missing genes/proteins [69]. This redundancy strongly complicates decision about the specific role in cold tolerance of a single gene/protein, or a group of gene/proteins, which are experimentally manipulated (*e*.*g*. by RNAi or by targetted mutation). In addition, Hsps up-regulation in *D*. *melanogaster* larvae was found to be related to long-term cold acclimation, during which the rates of many life processes are typically reduced and deep phenotypic change is induced [70]. Similarly as in the case of diapause-linked up-regulation of Hsps [71,72], the cold acclimation-linked up-regulation of Hsps might be related to regulation of developmental processes, apoptosis or immunity [73,74] rather than to repair of cold injury.

Considering the different reasons for inducible Hsps up-regulation, it is obvious that the cold-induced up-regulation response need not be either linked to, or necessary for, repair of direct cold injury in all situations, in agreement with the conclusion by Nielsen et al. [52]. For instance, we observed that larvae of both strains, Oregon and Hsp70^-^, dramatically increased cold tolerance in response to LTA (Fig 4, see also [14]), while only Oregon larvae were able to up-regulate the mRNA levels of *Hsp70*. Or another our observation: the mortality caused by chronic exposure (CE) to mild cold was similar in both strains in our study (Fig 4A) despite that only Oregon strain showed massive up-regulation of Hsp70 mRNA during recovery after the CE. Only in the case of severe acute cold shock, we have seen a difference in survival between the Oregon and Hsp70^-^ strain larvae.

### Up-regulation of *Hsp70* gene transcription might be required for the repair of cold injury caused by severe acute cold shock

We observed that survival is clearly compromised in Hsp70^-^ strain larvae in comparison to wild-type Oregon strain larvae when they are exposed to severe cold shocks (1-h-long) below -8°C (Fig 4B). The Oregon strain larvae had only 59.3%, 53.2% and 20.2% relative chance of death in comparison to Hsp70^-^ larvae when they were cold-shocked to -8°C, -10°C and -12°C, respectively. Interestingly, the survival rates after milder cold shocks of -2°C and -4°C were closely similar in both strains. These results suggest that protein denaturation caused by severe cold shocks may quantitatively and/or qualitatively differ from that caused by mild cold shocks. Therefore, the recruitment of Hsp70 in Oregon strain larvae might be needed to bolster the activity of other members of Hsps complex which then collectively help to repair cold injury resulting from severe cold shocks.

If the Hsp70 up-regulation has a positive effect on survival after severe cold shocks, it is reasonable to expect that survival will further improve after pre-stimulation of Hsp70 up-regulation using suitable pre-treatment. Based on similar principle, heat shock tolerance in *D*. *melanogaster* is clearly improved by preceding sub-lethal heat shocks [56,58,59] (see also Fig 3). In contrast to our expectation, we observed clearly negative effects of chronic or acute pre-treatments on survival after severe cold shock (Table 2). Similarly, the effects of pre-treatments on survival after chronic cold exposures were either neutral or weakly negative (Table 1). Again, the complexity of cold injury might help to explain such results: the application of pre-treatment always means increasing the total dose of cold and also mixing different types of cold injury (in the case of chronic pre-treatment *vs*. acute treatment and *vice versa*). Any potential positive effect of the pre-treatment on Hsps' up-regulation (linked to partial and often harmless protein denaturation) may be outweighed by negative effects that are linked to induction of stronger or additional cold injury.

### Gene expression response of the Hsps complex to cold

In this paper, we present detailed analysis of the changes in mRNA transcript abundance in response to cold in 24 different transcripts covering most of the Hsps complex in *D*. *melanogaster* plus four other genes that were previously related to repair of cold injury in the literature. The major aim of this analysis was to detect any potential compensation response for missing *Hsp70* gene in the Hsp70^-^ mutant larvae. No such compensation response was, however, detected. In contrast, the larvae of two strains, wild-type Oregon and mutant Hsp70^-^, exhibited closely similar patterns of gene expression in response to LTA and subsequently also to CE or CS (Figs 5 and 6). We are aware of limited power of gene transcription analysis to directly explain functional activity of respective gene products [75] and we also realize that the induction of synthesis of Hsps is partially regulated at the translational level [38]. The influence of *Hsp70* gene elimination on the heat-induced expression of other Hsps' coding genes was previously studied in Hsp70^-^ strain by Bettencourt et al. [59]. They concluded that although some alteration of both inducible and constitutive stress gene expression can be observed, it is ultimately insufficient to compensate for the loss of inducible tolerance of severe heat shock. Nevertheless, we expected to see, in the Hsp70^-^ strain, the cold-related transcriptional up-regulation in inducible *Hsps* (other than *Hsp70*) that could be driven by a common mechanism of the stress-regulated heat shock transcription factor, HSF [53,76].

In *Drosophila*, inactive HSF is present in monomeric form and/or is stabilized in complex with Hsps. Upon exposure to heat, cold or other stressors, HSF monomers and HSF-Hsps complexes are destabilized and become rapidly converted to trimeric forms [77]. The trimeric forms of HSF bind to the heat shock elements (HSEs) in inducible *Hsp*s' gene promoters and activate the transcription [78]. Because the significant part of HSEs is missing in the genome of Hsp70^-^ strain where 6 copies of *Hsp70* were deleted, we assumed that trimeric HSFs will bind remaining HSEs in other inducible *Hsp*s with higher frequency and, consequently, cause their "compensatory" up-regulation. We observed, however, that majority of typical HSF-regulated inducible *Hsp*s [39,53] exhibited closely similar temporal and magnitude patterns of up-regulation in both fly strains (S4 Fig). In the inducible *Hsp68*, the up-regulation in response to chronic exposure was even weaker in Hsp70^-^ strain than in Oregon strain (S4 Fig). On the other hand, the basal levels of mRNA transcripts of many inducible genes, including *Hsp68*, were constitutively higher in warm-acclimated larvae of Hsp70^-^ strain than in Oregon strain. Such constitutive up-regulation was not a result of "adaptation" in Hsp70^-^ strain to *Hsp70* deletion. It rather reflected genetic difference between Oregon and White strains [54] (see S3 Fig). Despite the constitutive up-regulation of several *Hsp*s genes in Oregon strain, the larvae of Oregon and White strains showed nearly identical levels of heat tolerance, including almost identical responses to pre-treatment at sub-lethal dose of heat (compare our results with Oregon strain, Fig 3, to results with White strain [58,59]). Therefore, we assume that the constitutive up-regulation of inducible Hsps' mRNAs in Hsp70^-^ strain had negligible effect on both heat- and cold-tolerance.

The central role of HSF in transcriptional up-regulation of inducible Hsps is well described [53,76,79]. In *D*. *melanogaster*, four alternatively spliced *Hsf* isoforms (*A*, *B*, *C*, *D*) were identified, and the ratio of these isoforms was shown to be regulated by heat/cold stress. The relative proportion of isoform *Hsf B* increased approximately 2.5-fold upon heat exposure (37°C/1 h), while that of *Hsf D* increased approximately 5-fold upon cold exposure (4°C/2 h) in adult flies [80]. We extended this earlier observation and found that LTA was accompanied with drastic down-regulation of isoforms *Hsf A* (more than 130-fold) and *Hsf B* (10-fold), while the isoforms *Hsf C* and *Hsf D* were strongly up-regulated (approximately 5-fold and more than 80-fold, respectively) (S4 Fig). The transcriptional activities of different HSF isoforms were studied using reporter assay and it was found that the isoforms C and D exhibit several-fold greater transcriptional activities than the isoforms A and B [80]. Thus, it is possible that alternative splicing helps tuning the sensitivity of Hsps up-regulation response to environmental temperature. When the environmental temperature decreases (*i*.*e*. during LTA), the isoforms with higher transcriptional activities (C and D) may be preferentially recruited in order to compensate for lower rates of biochemical reactions at lower temperatures. When the environmental temperature increases, the high proportion of low transcriptional activity isoforms (A and B) may help to protect the organism against deleterious consequences of *Hsp70* overexpression [81,82]. In addition, Fujikake et al. [80] suggested that different isoforms might induce different Hsps. This hypothesis remains untested. Nevertheless, the temperature- regulated alternative splicing and dramatic up-regulation of specific isoforms at cold brings additional piece of indirect support for the view that the Hsps complex has a vital role at low body temperatures in *D*. *melanogaster*.

### Responses of other stress-related genes to cold

Product of gene *Frost* has unknown function but it was suggested as a candidate cold tolerance-mediating gene based upon frequent observations of its mRNA up-regulation in response to cold [49, 83–85]. We observed that *Fst* mRNA levels increased 2–4-fold in larvae of two strains of *D*. *melanogaster* in response to LTA. In contrast to earlier studies, however, no further up-regulation in response to either CE or CS was observed and the *Fst* mRNA gradually returned to pre-acclimation levels during recovery (S4 Fig). Silencing the *Fst* expression by RNAi increased the recovery time from chill-coma [86], but survival of 2 h exposures to sub-zero temperatures in *Fst* RNAi lines was not lower than that in a control line [87].

Menin has been implicated to play a role in control of gene expression [88]. Other study [89] indicated that Menin is involved in the regulation of inducible Hsps expression. The over-expression of *Mnn* enhanced the expression of *Hsp70* in *D*. *melanogaster* embryos, while the RNAi inhibition of *Mnn* reduced the expression of *Hsp70* and blocked the activation of *Hsp23* upon heat shock. That is why we assessed the mRNA levels of *Mnn* and found them increasing approximately 5-fold in response to LTA in Hsp70^-^ strain, while only 1.8-fold in Oregon strain. Further up-regulation, approximately 4-fold, of *Mnn* levels was observed in response to CS but only in the Oregon strain (S4 Fig). Potential linkage between *Mnn* expression and the mRNA levels of seven small *Hsp*s was indicated by their unique two-step response to LTA, where the mRNA levels decreased at 15°C, while the mRNA levels increased in response to final step of LTA at 6°C/2 d (S4 Fig).

The members of a family of cold shock domain-containing proteins are known to be induced in response to cold shock in bacteria and their role is believed to be binding mRNA and regulation of ribosomal translation at low temperatures [90,91]. The cold shock domain also occurs in so called Cold shock protein in *Drosophila melanogaster* but its function is not fully understood. Almost no significant changes in *Csp* mRNA levels were seen in response to cold in two strains of *D*. *melanogaster* (S4 Fig).

Starvin is a sole member of BAG family in *D*. *melanogaster* [92]. The BAG (Bcl-2-associated anthanogene) proteins bind the ATPase domain of Hsp70 and may thus regulate its chaperoning activity [93]. Colinet and Hoffmann [94] showed that the cold exposure (0°C/9 h) up-regulates *Stv* mRNA abundance approximately 8-fold, while the protein levels increase approximately 2.5-fold in adult fruit flies. The temporal patterns of cold-induced expression were closely similar in *Stv* and *Hsp70* genes, which supported the view that Starvin may act as co-chaperone which regulates the activity of Hsp70 during recovery from cold stress [94]. In accordance with these results, we observed approximately 3-4-fold up-regulation of *Stv* mRNA levels in response to LTA and further 2-3-fold up-regulations in response to CE and CS in both fly strains (S4 Fig).

## Conclusions

In conclusion, this study suggests that cold-induced up-regulation of a whole complex of inducible Hsps genes in *D*. *melanogaster* larvae might be linked to alternative splicing of *Hsf* mRNA, which favors production of splice variants C and D over the production of splice variants A and B at low but above-zero environmental temperatures (during long-term cold-acclimation). The exposure to cold, either chronic mild cold (0°C for different periods of time) or acute cold shocks (different sub-zero temperatures for fixed period of time of 1 h), results in further up-regulation of inducible Hsps, including massive mRNA expression from *Hsp70* gene loci. The Hsp70^-^ null mutant of *D*. *melanogaster* shows no compensation (for missing *Hsp70* gene) of the cold-induced up-regulation response at the mRNA level. Despite that, the survival of Hsp70^-^ null mutant larvae is not impaired in comparison to wild-type strain larvae when they are exposed either to chronic cold or to mild cold shocks. The cold tolerance in Hsp70^-^ null mutants is compromised only when the larvae are exposed to severe cold shocks of or below -8°C. This suggests that mild and severe cold shocks might result in different types of cold injury expressed as quantitative and/or qualitative difference in the protein cold-denaturation. The recruitment of inducible Hsp70 might be needed to bolster the activity of other members of Hsps complex, which then collectively help to cope with denatured proteins produced by severe cold shocks.

## Supporting Information

S1 Fig Absence of *Hsp70* gene sequence in the gDNA extracted from Hsp70^-^ strain. (DOCX)

S2 Fig Relative stability of mRNA levels in reference genes. (DOCX)

S3 Fig Constitutive levels of expression in target genes. (DOCX)

S4 Fig The patterns of gene expression in response to experimental treatments. (DOCX)

S1 Table List of target genes and gene specific oligonucleotide primers used for qRT-PCR. (DOCX)
