# Supplementary material for: The Role of Inducible Hsp70, and Other Heat Shock Proteins, in Adaptive Complex of Cold Tolerance of the Fruit Fly (Drosophila melanogaster)
Source: PLoS One. 2015 Jun 2;10(6):e0128976. doi: 10.1371/journal.pone.0128976 (PMC4452724; doi:10.1371/journal.pone.0128976)
Supplement: S1 Fig — (DOCX) [file pone.0128976.s001.docx]

**The role of inducible Hsp70, and other heat shock proteins, in adaptive complex of cold tolerance of the fruit fly (*Drosophila melanogaster*).**

**Supporting Information Figure S1:**

Absence of *Hsp70* gene sequence in the gDNA extracted from Hsp70- strain.


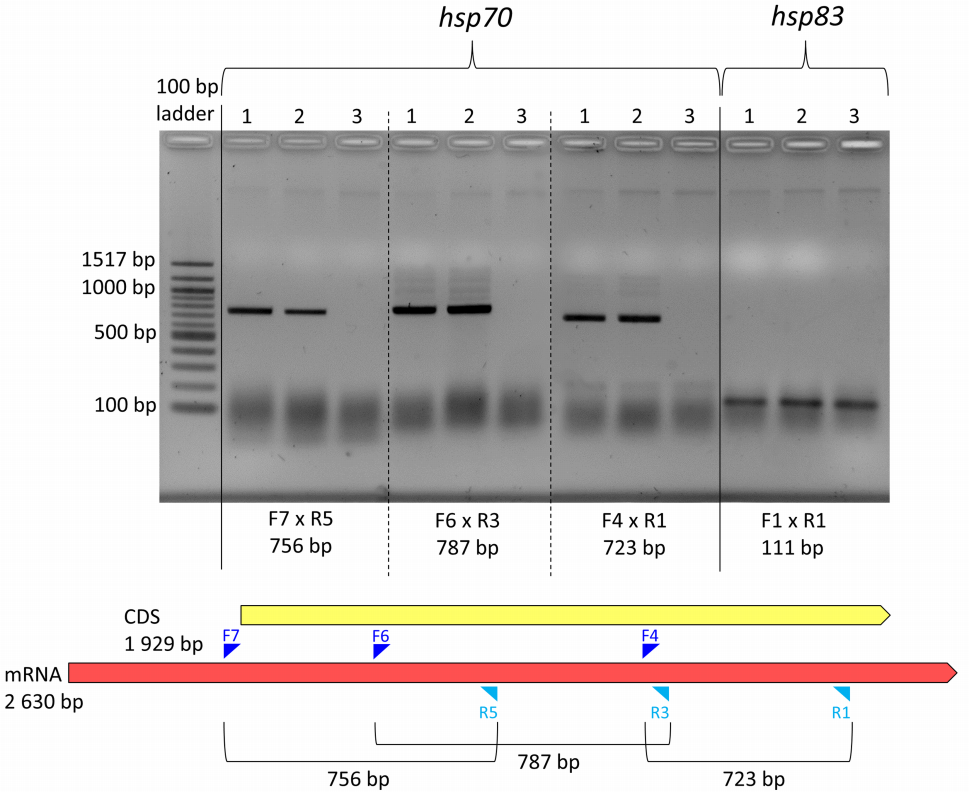


PCR amplifications using three different *hsp70*-specific oligonucleotide primers pairs (see Table below) produced unique PCR products of expected size when genomic DNA extracted from Oregon and White strains of *D. melanogaster* was used as PCR template. In contrast, *Hsp70*-specific products were never observed in reactions using genomic DNA extracted from Hsp70^-^ strain flies as a template. Using the primers for *hsp83* gene as positive control, the amplicons of expected size were produced from gDNAs' of all three strains. These results confirm that sequences coding for *Hsp70* gene are not present in the genome of Hsp70^-^ strain flies.

**Table:** Gene specific oligonucleotide primers used for verification of the presence/absence

of *Hsp70* gene in the genomes of three strains of *Drosophila melanogaster*.

| Gene | Primer name and direction | Sequence (5‘ → 3‘) | Amplicon size (bp) |
| --- | --- | --- | --- |
| *Hsp70* | F7 Fwd | ccgaggaagaagaactcaca | 756 |
|  | R5 Rev | cttgaactcgtccgccagatgag |  |
|  | F6 Fwd | ggcatatctgggcgagagcatc | 787 |
|  | R3 Rev | cagcagcacgtcctggatcttgcc |  |
|  | F4 Fwd | ctcagcggagaccagagcggcaag | 723 |
|  | R1 Rev | tcttggtcatgatgggggagcagtg |  |
| *Hsp83* | F1 Fwd | gcacgccagccgcatctacc | 111 |
|  | R1 Rev | tcaaccagcgagggggcatct |  |
