# Supplementary material for: The Role of Inducible Hsp70, and Other Heat Shock Proteins, in Adaptive Complex of Cold Tolerance of the Fruit Fly (Drosophila melanogaster)
Source: PLoS One. 2015 Jun 2;10(6):e0128976. doi: 10.1371/journal.pone.0128976 (PMC4452724; doi:10.1371/journal.pone.0128976)
Supplement: S2 Fig — (DOCX) [file pone.0128976.s002.docx]

**The role of inducible Hsp70, and other heat shock proteins, in adaptive complex of cold tolerance of the fruit fly (*Drosophila melanogaster*).**

**Supporting Information Figure S2:**

Relative stability of mRNA levels in reference genes.

The levels of mRNA transcripts of reference genes (estimated as *C*_Q_ values in qRT-PCR analysis) were relatively stable over our experimental treatments. Small variations in *C*_Q_ values were caused partially by random fluctuations due to technical limits of our qRT-PCR analysis, and partially by systematic transcriptional responses of the reference genes to treatments. We decided to neglect any potential systematic responses of reference genes in our statistical analysis because the patterns were not obvious (not separable for random fluctuations) and also because the overall variation in *C*_Q_ values in reference genes was ± 0.67 cycles in maximum (corresponding to ± 1.42-fold change max), while the fold-changes observed in mRNA transcripts of highly regulated target genes were often much higher, sometimes two orders in magnitude.
