# Supplementary material for: The Role of Inducible Hsp70, and Other Heat Shock Proteins, in Adaptive Complex of Cold Tolerance of the Fruit Fly (Drosophila melanogaster)
Source: PLoS One. 2015 Jun 2;10(6):e0128976. doi: 10.1371/journal.pone.0128976 (PMC4452724; doi:10.1371/journal.pone.0128976)
Supplement: S3 Fig — (DOCX) [file pone.0128976.s003.docx]

**The role of inducible Hsp70, and other heat shock proteins, in adaptive complex of cold tolerance of the fruit fly (*Drosophila melanogaster*).**

**Supporting Information Figure S3:**

Constitutive levels of expression in target genes.

**
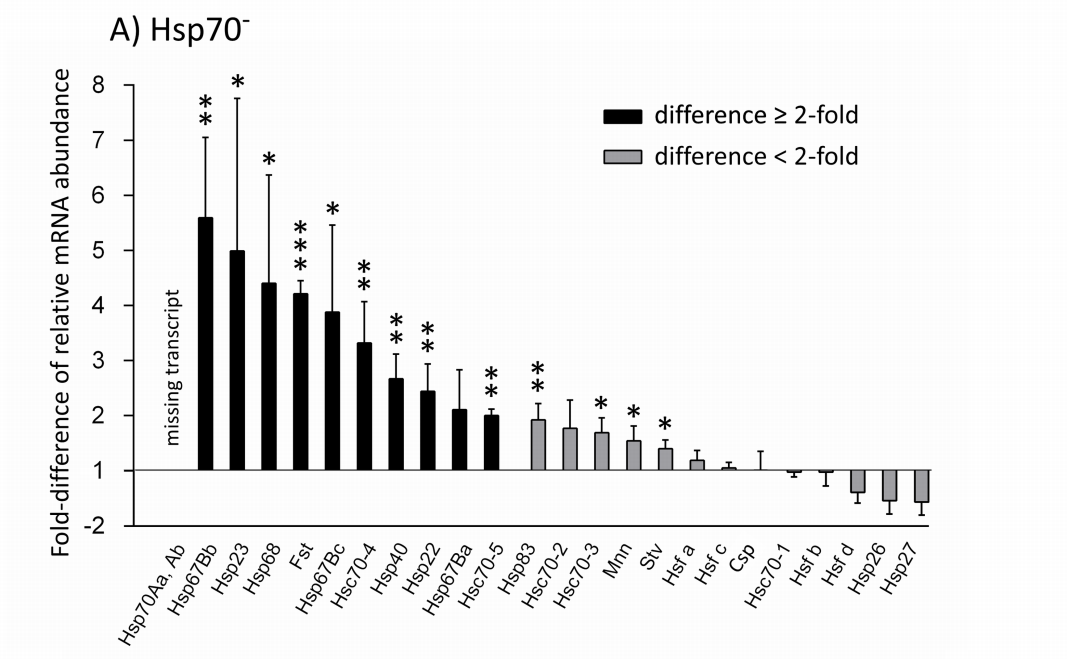
**


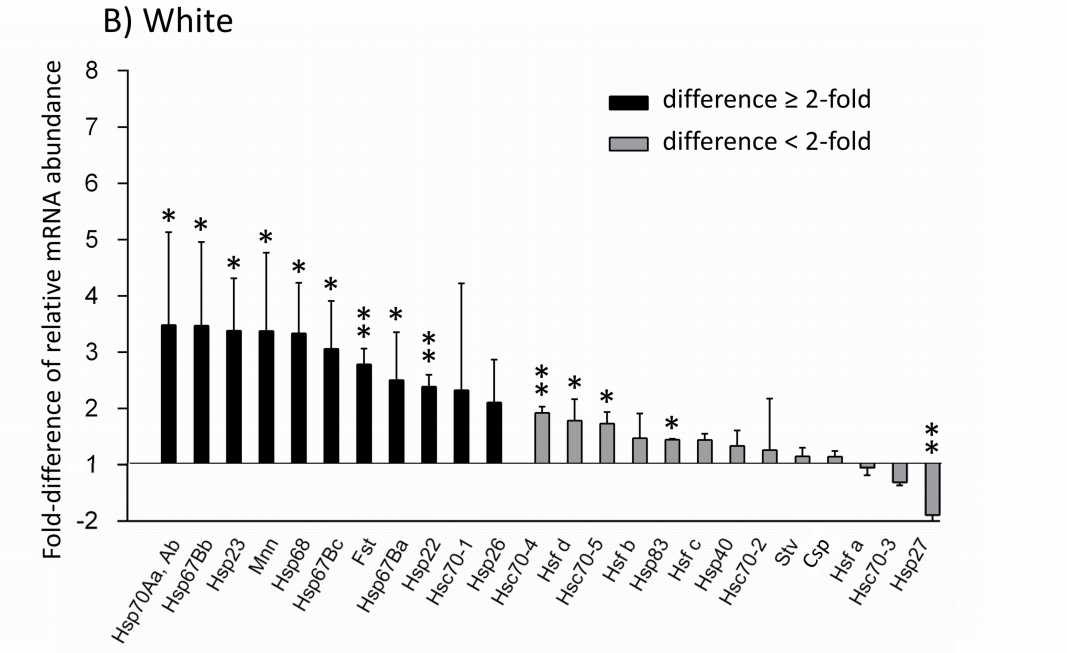


The columns show fold-differences in the relative abundance of mRNA transcripts of target genes (*x* axis) between (A) the Hsp70^-^ strain larvae or (B) the White strain larvae and the Oregon strain larvae (the levels were normalized to 1 in Oregon strain) that were acclimated at constant 25°C. Statistical significance of the differences was assessed using Student's unpaired two-tailed t-tests (*, *P* < 0.05; **, P < 0.01; ***, *P* < 0.001).
