# Supplementary material for: The Role of Inducible Hsp70, and Other Heat Shock Proteins, in Adaptive Complex of Cold Tolerance of the Fruit Fly (Drosophila melanogaster)
Source: PLoS One. 2015 Jun 2;10(6):e0128976. doi: 10.1371/journal.pone.0128976 (PMC4452724; doi:10.1371/journal.pone.0128976)
Supplement: S4 Fig — (DOCX) [file pone.0128976.s004.docx]

**The role of inducible Hsp70, and other heat shock proteins, in adaptive complex of cold tolerance of the fruit fly (*Drosophila melanogaster*).**

**Supporting Information Figure S4:**

The patterns of gene expression in response to experimental treatments.

The columns show abundance of mRNA transcripts of target genes relative to abundance of mRNA transcripts of two reference genes (*Rpl32* and *β-tubulin*). Each column is a mean ± SD of qRT-PCR analysis of three biological replications. The values (fold-differences) are normalized to 1 = mean relative mRNA level in the larvae acclimated to constant 25°C (acclimation protocol A). The results for two *D. melanogaster* strains, Oregon and Hsp70^-^, are presented side by side for each gene.

The statistical analysis was performed on Log2-transformed values. Three independent one-way ANOVAs followed by Bonferroni post-hoc tests were applied: first, to test the influence of acclimation protocol on gene expression (means flanked with different letters are statistically different); second, to assess the gene expression during recovery from CE; and, third, to assess the gene expression during recovery from CS. In the latter two cases, the differences between the initial value (acclimation C) and recovery-related values were tested (*, *P* < 0.05; **, P < 0.01; ***, *P* < 0.001).

White columns represent three different acclimation protocols:

(A) constant 25°C;

(B) constant 15°C (this protocol was not applied to Hsp70^-^ strain larvae);

(C) constant 15°C followed by 2 days at constant 6°C.

Grey columns represent three times (1, 3, 24 h) sampled during recovery after chronic cold exposure (CE) to 0°C/1.25 d.

Black columns represent three times (1, 3, 24 h) sampled during recovery after acute cold shock (CS) to -4°C/1 h.
