# Supplementary material for: The Role of Inducible Hsp70, and Other Heat Shock Proteins, in Adaptive Complex of Cold Tolerance of the Fruit Fly (Drosophila melanogaster)
Source: PLoS One. 2015 Jun 2;10(6):e0128976. doi: 10.1371/journal.pone.0128976 (PMC4452724; doi:10.1371/journal.pone.0128976)
Supplement: S1 Table — (DOCX) [file pone.0128976.s005.docx]

**The role of inducible Hsp70, and other heat shock proteins, in adaptive complex of cold tolerance of the fruit fly (*Drosophila melanogaster*).**

**Supporting Information Table S1:**

List of target genes and gene specific oligonucleotide primers used for qRT-PCR

(A) Inducible Heat shock genes

| Gene | | | Primer  direction | Sequence  (5‘ → 3‘) | Amplicon  size (bp) | PCR product blastn 1st hit * | | | PCR product blastn 2nd hit * | | |
| --- | --- | --- | --- | --- | --- | --- | --- | --- | --- | --- | --- |
| Name | FlyBase ID | Abbreviation |  |  |  | Abbr. | Score | E value | Abbr. | Score | E value |
| *Heat shock proteins*  *70Aa, Ab* | FBgn0013275  FBgn0013276 | *Hsp70Aa, Ab* | Fwd | ggcatatctgggcgagagcatc | 346 | Hsp70Aa, Ab, Ba, Bb, Bbb, Bc | 420.752  –  446.523 | 4.496e-117 –  7.854e-125 | Hsp68 | 163.046 | 1.6985e-39 |
|  |  |  | Rev | cttgaactcgtccgccagatgag |  |  |  |  |  |  |  |
| *Heat shock protein 68* | FBgn0001230 | *Hsp68* | Fwd | atcgccgcccgcaatcaactgg | 169 | Hsp68 | 222.517 | 9.8358e-58 | Hsp70Bc | 65.9106 | 1.3674e-10 |
|  |  |  | Rev | actcgtactcctccttctcggcggtgg |  |  |  |  |  |  |  |
| *Heat shock protein 83* | FBgn0001233 | *Hsp83* | Fwd | gcacgccagccgcatctacc | 111 | Hsp83 | 107.54 | 2.4893e-23 | CG13868 | 34.1929 | 0.299065 |
|  |  |  | Rev | tcaaccagcgagggggcatct |  |  |  |  |  |  |  |
| *Heat shock gene 67Ba* | FBgn0001227 | *Hsp67Ba* | Fwd | cagccgccaaagccgtcagaggtaga | 111 | Hsp67Ba | 63.9282 | 2.4195e-10 | Ubi-p5E | 32.2105 | 0.854472 |
|  |  |  | Rev | ggcgagagtgagcgagagcgaaggagt |  |  |  |  |  |  |  |
| *Heat shock gene 67Bb* | FBgn0001228 | *Hsp67Bb* | Fwd | tgaactggacaaggctctaaatctgga | 163 | Hsp67Bb-RA/B/C/D  Hsp22-RA | 184.852 | 1.9389e-46 | Mrp4-RB | 30.2282 | 6.82187 |
|  |  |  | Rev | caccacattgctgtatccctgacttttg |  |  |  |  |  |  |  |
| *Heat shock gene 67Bc* | FBgn0001229 | *Hsp67Bc ^#^* | Fwd | agccgcggtggagcctcaaataag | 142 | Hsp67Bc | 125.381 | 1.0111e-28 | Hsp27 | 40.14 | 0.0046238 |
|  |  |  | Rev | gatcgtcctcgcgctcctcgtgtt |  |  |  |  |  |  |  |
| *Heat shock protein 22* | FBgn0001223 | *Hsp22 ^#^* | Fwd | ctctcctcgccctttcacgccttcttc | 145 | Hsp22-RA/B  Hsp67Bb-RB | 143.222 | 4.5841e-34 | IntS10 | 34.1929 | 0.303666 |
|  |  |  | Rev | gggtgagtttgtagccatccttgttgacg |  |  |  |  |  |  |  |
| *Heat shock protein 23* | FBgn0001224 | *Hsp23* | Fwd | tgcgccagctggagaaaca | 238 | Hsp23 | 341.458 | 2.3782e-93 | Hsp27 | 77.8047 | 5.5429e-14 |
|  |  |  | Rev | tcataaccgggtggcagagc |  |  |  |  |  |  |  |
| *Heat shock protein 26* | FBgn0001225 | *Hsp26* | Fwd | ccaggagccccgcagccccatctacg | 172 | Hsp26 | 214.587 | 2.5804e-55 | salm | 32.2105 | 2.05357 |
|  |  |  | Rev | catctcgcgccgcaaagccaaaacctg |  |  |  |  |  |  |  |
| *Heat shock protein 27* | FBgn0001226 | *Hsp27* | Fwd | atttgctggaggatgacttcggttttg | 132 | Hsp27 | 117.452 | 2.1469e-26 | CG1358 | 32.2105 | 0.981733 |
|  |  |  | Rev | ggtggccatggctcctctcgta |  |  |  |  |  |  |  |
| *DnaJ-like-1/*  *Hsp40* | FBgn0263106 | *Hsp40* | Fwd | gcagggcagcaggatacaggtgaa | 199 | DnaJ-1 | 295.864 | 1.0916e-79 | chm | 34.1929 | 0.64389 |
|  |  |  | Rev | ctagttgggcagcagctcggacag |  |  |  |  |  |  |  |

(B) Heat shock cognates

| Gene | | | Primer  direction | Sequence  (5‘ → 3‘) | Amplicon  size (bp) | PCR product blastn 1st hit * | | | PCR product blastn 2nd hit * | | |
| --- | --- | --- | --- | --- | --- | --- | --- | --- | --- | --- | --- |
| Name | FlyBase ID | Abbreviation |  |  |  | Abbr. | Score | E value | Abbr. | Score | E value |
| *Heat shock cognate 1* | FBgn0001216 | *Hsc70-1* | Fwd | gagataacaacagcctgggtaag | 749 | Hsc70-1 | 995.635 | 0 | Hsc70-4 | 77.8047 | 1.6861e-13 |
|  |  |  | Rev | ctcaatggttggacccgag |  |  |  |  |  |  |  |
| *Heat shock cognate 2* | FBgn0001217 | *Hsc70-2* | Fwd | tccgaaaccgaacgctccaagg | 113 | Hsc70-2 | 91.6812 | 9.7865e-19 | Hsp70Aa, Ab, Ba, Bb, Bbb, Bc | 34.1929 | 0.197843 |
|  |  |  | Rev | tggcactctttcaggtgactc |  |  |  |  |  |  |  |
| *Heat shock cognate 3* | FBgn0001218 | *Hsc70-3* | Fwd | accagaaccgcctgacgccc | 254 | Hsc70-3 | 339.475 | 1.0847e-92 | CG14515 | 42.1223 | 0.00352804 |
|  |  |  | Rev | gggatcggcatcgggattctgt |  |  |  |  |  |  |  |
| *Heat shock cognate 4* | FBgn0266599 | *Hsc70-4* | Fwd | tgccctggagcgttcgaccaa | 419 | Hsc70-4 | 186.834 | 6.1982e-47 | Dscam4 | 38.1576 | 0.0353483 |
|  |  |  | Rev | gcatacctccgggaccgcc |  |  |  |  |  |  |  |
| *Heat shock cognate 5* | FBgn0001220 | *Hsc70-5* | Fwd | ataagcttctcggctcctttaca | 285 | Hsc70-5 | 438.593 | 1.864e-122 | Hsc70-4 | 42.1223 | 0.0041695 |
|  |  |  | Rev | tatcgtggactatgctctcgccc |  |  |  |  |  |  |  |

(C) Splicing variants of Heat shock factor**

| Gene | | | Primer  direction | Sequence  (5‘ → 3‘) | Amplicon  size (bp) | PCR product blastn 1st hit * | | | PCR product blastn 2nd hit * | | |
| --- | --- | --- | --- | --- | --- | --- | --- | --- | --- | --- | --- |
| Name | FlyBase ID | Abbreviation |  |  |  | Abbr. | Score | E value | Abbr. | Score | E value |
| *Heat shock*  *factor* | FBgn0001222 | *Hsf A* | Fwd | ctcctcaggatggcctcagttgacg | 153 | Hsf-RA/B | 139.258 | 9.973e-33 | Hsf-RC/D | 99.6106 | 8.5859e-21 |
|  |  |  | Rev | gtccattagatcagagtcgttaaacagaccca |  |  |  |  |  |  |  |
|  |  | *Hsf B* | Fwd | ccataatcaagcggctaattcttactgttgcag | 166 | Hsf-RA/B | 190.799 | 3.0104e-48 | Hsf-RC/D | 135.293 | 1.5401e-31 |
|  |  |  | Rev | gtccattagatcagagtcgttaaacagaccca |  |  |  |  |  |  |  |
|  |  | *Hsf C* | Fwd | gtccattagatcagagtcgttaaacagaccca | 148 | Hsf-RC/D | 155.117 | 1.2226e-37 | Hsf-RA/B | 111.505 | 1.6434e-24 |
|  |  |  | Rev | aattgggcgtcctgctaagcgcacc |  |  |  |  |  |  |  |
|  |  | *Hsf D* | Fwd | ccataatcaagcggctaattcttactgttgcag | 161 | Hsf-RC/D | 170.975 | 2.4249e-42 | Hsf-RA/B | 127.364 | 3.2595e-29 |
|  |  |  | Rev | aattgggcgtcctgctaagcgcacc |  |  |  |  |  |  |  |

(D) Miscellaneous genes

| Gene | | | Primer  direction | Sequence  (5‘ → 3‘) | Amplicon  size (bp) | PCR product blastn 1st hit * | | | PCR product blastn 2nd hit * | | |
| --- | --- | --- | --- | --- | --- | --- | --- | --- | --- | --- | --- |
| Name | FlyBase ID | Abbreviation |  |  |  | Abbr. | Score | E value | Abbr. | Score | E value |
| *Frost* | FBgn0037724 | *Fst* | Fwd | cgactctgaggatggtagcggttctga | 223 | Fst | 119.434 | 1.2069e-26 | NFAT | 36.1753 | 0.139674 |
|  |  |  | Rev | ggattcttcgggagcctcggtcgtc |  |  |  |  |  |  |  |
| *Menin 1* | FBgn0031885 | *Mnn* | Fwd | gcttcggcgttgctctggctgtggtg | 171 | Mnn1 | 184.852 | 1.8980e-46 | CG3106 | 34.1929 | 0.427727 |
|  |  |  | Rev | tatcctgcccccgcttatcctcgcttcctt |  |  |  |  |  |  |  |
| *Starvin* | FBgn0086708 | *Stv* | Fwd | cggtcgctcggaggttgattgtgc | 135 | Stv | 143.222 | 4.5841e-34 | CG34398 | 34.1929 | 0.303666 |
|  |  |  | Rev | tctggcggaggggcggagtgg |  |  |  |  |  |  |  |
| *Cold shock protein* | FBgn0036661 | *Csp* | Fwd | gctcctcgcccgctaagtaaag | 258 | CG9705 | 119.434 | 7.0434e-27 | CG45072 | 36.1753 | 0.0815083 |
|  |  |  | Rev | ggcaggcctgttgttccattctatt |  |  |  |  |  |  |  |

(E) Reference genes

| Gene | | | Primer  direction | Sequence  (5‘ → 3‘) | Amplicon  size (bp) |
| --- | --- | --- | --- | --- | --- |
| Name | FlyBase ID | Abbreviation |  |  |  |
| *β-Tubulin at 56D* | FBgn0003887 | *βTub* | Fwd | acatcccgccccgtggtctgaa | 257 |
|  |  |  | Rev | ctgctcctcctcgaactcggcgtc |  |
| *Ribosomal*  *protein L32* | FBgn0002626 | *RpL32* | Fwd | atgctaagctgtcggtga | 202 |
|  |  |  | Rev | gaatccggtgggcagcat |  |

* The specificity was assayed in all primer pairs by sequencing their respective PCR products. The identity of each PCR product was validated by nucleotide blast (blastn) analysis (NCBI).

** The primer sequences were designed according to: Fujikake N, Nagai Y, Popiel HA, Kano H, Yamaguchi M, Toda T (2005) Alternative splicing regulates the transcriptional activity of *Drosophila* heat shock transcription factor in response to heat/cold stress. FEBS Letters 579: 3842-3848.

# The genes *Hsp67Bb* and *Hsp22* are transcribed from the same gDNA locus (green bars) and some of their alternative mRNA transcripts overlap (red bars). We designed the primers inside the coding sequences (CDS, yellow bars) of both respective genes in order to ensure that they prime amplification of PCR products from the most relevant mRNA transcripts. Nevertheless, the primer pairs were not perfectly specific for their respective target genes and probably were also priming the PCR products from less frequent mRNA transcripts of the other gene.

Since the gene expression profiles differed in *Hsp67Bb* and *Hsp22* genes, we decided to keep the data in the paper.


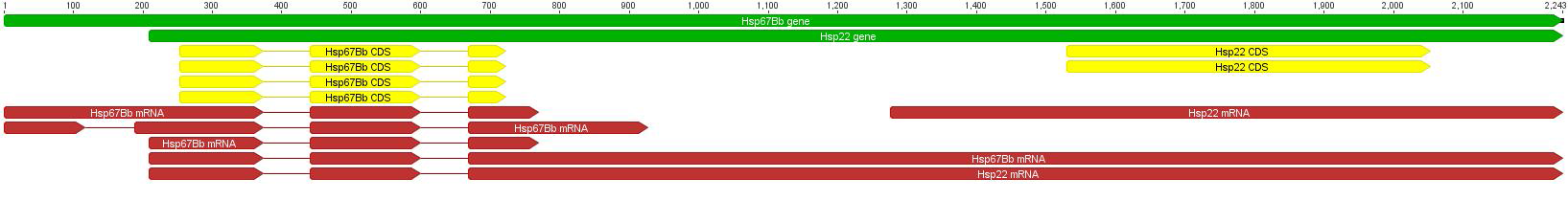


*Hsp67Bb* Rev

*Hsp22* Fwd

*Hsp22* Rev

*Hsp67Bb* Fwd
